# Correlation Analyses between Histological Staging and Molecular Alterations in Tumor-Derived and Cell-Free DNA of Early-Stage Primary Cutaneous Melanoma

**DOI:** 10.3390/cancers15215141

**Published:** 2023-10-25

**Authors:** Szilvia Lilla Csoma, Kristóf Madarász, Yi Che Chang Chien, Gabriella Emri, Judit Bedekovics, Gábor Méhes, Attila Mokánszki

**Affiliations:** 1 Department of Pathology, Faculty of Medicine, University of Debrecen, H-4032 Debrecen, Hungary; csoma.szilvia@med.unideb.hu (S.L.C.); madarasz.kristof@med.unideb.hu (K.M.); dr.changchien.yiche@med.unideb.hu (Y.C.C.C.); bedekovics.judit@med.unideb.hu (J.B.); gabor.mehes@med.unideb.hu (G.M.); 2 Department of Dermatology, Faculty of Medicine, University of Debrecen, H-4032 Debrecen, Hungary; gemri@med.unideb.hu

**Keywords:** primary cutaneous melanoma, Breslow depth, Clark level, somatic variant allele frequency, cell-free DNA, *BRAF* gene mutation, digital PCR

## Abstract

**Simple Summary:**

Primary cutaneous melanoma (PCM) is a highly aggressive and potentially lethal form of skin neoplasm with a rapidly increasing incidence rate worldwide. The most common genetic aberration in PCM is the *BRAF* gene p.V600E pathogenic variant. The use of liquid biopsy (LB), which is a non-invasive, low-risk procedure that can be repeated multiple times, is becoming increasingly important in precision oncology. Because of the limited information about the applicability of LB in melanoma, here we investigate the correlation analyses and statistical significance between histopathological staging and molecular alterations in tumor-derived and cell-free DNA. The Breslow depth (BD) and Clark level were applied to categorize the study population. A positive correlation was proven between the tumor depth and peripheral blood plasma cfDNA yield in all mutant and negative cases. This observation is also supported by the fact that a statistically significantly higher concentration of cfDNA can be isolated from Clark V category cases compared to the others.

**Abstract:**

Here, we investigate the correlation and statistical analyses between histological staging and molecular alterations in tumor-derived (tdDNA) and cell-free DNA (cfDNA) obtained from early-stage primary cutaneous melanoma (PCM) patients using digital PCR (dPCR) for the detection of the *BRAF* p.V600E somatic pathogenic variant. In the prospective study, a total of 68 plasma and paired tdDNA samples, and in the retrospective cohort, a total of 100 tdDNA samples were analyzed using dPCR and reverse hybridization StripAssay. The Breslow depth (BD) and Clark level were applied to categorize the study population. Our results demonstrate that dPCR is a highly sensitive and specific method for the detection of *BRAF* p.V600E somatic variants in cfDNA samples from PCM patients. A strong correlation was detected between BD and cfDNA concentration in all mutant and negative cases, between the tdDNA concentration and the tumor-derived variant allele frequency (VAF) of *BRAF* p.V600E, between the tdVAF and the cfVAF in all cases, and between the cfDNA and cfVAF in mutant cases. The tdVAF and cfVAF of *BRAF* p.V600E and cfDNA concentration were the highest in Clark’s V category. The cfDNA concentration was statistically significantly higher in Clark’s III, IV, and V groups compared to cases with a better prognosis. It can also be explained by the fact that cases with a more advanced stage classification release more cfDNA into the peripheral circulation.

## 1. Introduction

Primary cutaneous melanoma (PCM) is a highly aggressive and potentially lethal form of skin neoplasm with a rapidly increasing incidence rate worldwide [1,2]. It is the most dangerous form of skin cancer because it can give distant metastasis if not detected and treated early. It accounts for only about 1% of all skin neoplasms; however, it causes the majority of fatal consequences [3]. Early detection of PCM is essential for effective treatment and improved patient outcomes. Treatment options include surgical removal of the total tumor area, radio-, chemo-, immune- and/or targeted therapy [4,5,6]. In the area of modern personalized oncology, immune- and targeted therapy underwent rapid development and became the focus of tumor management.

The most common genetic aberration in PCM affects the *BRAF* gene [7,8]. The gene is located on chromosome 7 and consists of 6459 bp and 18 exons. The BRAF protein consists of 766 amino acids and has a RAS binding domain, a cysteine-rich domain, a kinase domain, and three conserved regions [9,10,11]. The BRAF protein is a serine/threonine kinase that is a member of the RAF serine/threonine protein kinase enzyme family. This family includes three kinases, ARAF, BRAF, and CRAF/RAF1, of which the BRAF protein has the highest kinase activity. Active BRAF leads to the serial activation of transcription factors, which plays a role in many biological processes, such as cell differentiation, proliferation, growth, and apoptosis [12,13,14,15,16]. In total, 90% of *BRAF* genetic aberrations are caused by a somatic point mutation, which is thymine to adenine transversion at nucleotide position 1799. This missense mutation occurs in exon 15 of the *BRAF* gene and results in the replacement of valine in codon 600 with glutamic acid (*BRAF* c.1799T>A; p.V600E). This pathogenic variant enhances the constitutive activation of the mitogen-activated protein kinase pathway, thereby enabling the activation of the signaling cascade even in the absence of an extracellular signal [9,17].

Molecular diagnosis of cancer patients is primarily based on tissue samples, which can have significant limitations. Individual tumors are genetically heterogeneous, and the small amount of tissue obtained through needle biopsy may not necessarily represent the most aggressive subclones. Moreover, several cancer types, such as certain types of lung cancer, are located in anatomically challenging areas for needle biopsy, making the sampling difficult and risky. These challenges have led to the development of a new diagnostic concept called liquid biopsy (LB) [18]. In oncology, this term is used in a broad sense, referring to the sampling and analysis of various biological fluids, primarily blood, but also relatively accessible body fluids such as urine or ascites [19]. The use of LB, which is a non-invasive, low-risk procedure that can be repeated multiple times, is becoming increasingly important in precisional oncology. It allows for longitudinal monitoring of the therapy follow-up of patients, provides information about the dynamics of genetic alterations at different stages of tumorigenesis, and can be used for early diagnosis of malignant diseases. LB examines circulating tumor cells and circulating nucleic acids (cfDNA), including circulating tumor DNA (ctDNA) fragments, which are found in peripheral blood (PB) and other body fluids [20,21,22].

Limited information about the applicability of LB in melanoma is available in clinical practice because the size of the PCMs is relatively small; it is outside on the body surface, and therefore, a small amount of cfDNA is derived from it [23]. Due to the anatomical peculiarities of melanomas, ultra-sensitive methods are needed for the molecular analysis of cfDNA. The diagnostic utility is limited by the analytical sensitivity of the methods used for the detection of somatic variants in this form of nucleic acids. The presence of specific genetic alterations, such as the *BRAF* p.V600E somatic variant, has significant diagnostic and prognostic implications in PCM. Aberrations with extremely low allele frequencies, especially in LB samples, are best identified with digital PCR (dPCR) [24]. Digital PCR is a powerful technique that enables the detection and quantification of nucleic acids with unparalleled precision and sensitivity. In contrast to the traditional PCR reaction, during digital PCR, the starting nucleic acid molecules are amplified in separate reaction spaces, and only one or a few DNA molecules are found in each reaction space. The initial copy number and the density of the searched sequences can be calculated using the Poisson distribution based on the positive reactions [25,26]. It has emerged as a promising tool for the detection of somatic variants in cfDNA samples, offering a non-invasive and reliable alternative to traditional diagnostic methods. The currently available dPCR platforms involve droplet- (Bio-Rad, Hercules, CA, USA), chip- (Thermo Fisher, Waltham, MA, USA), and nanoplate-based workflows (Qiagen, Hilden, Germany). Due to the high sensitivity of the dPCR technique, it is suitable for the detection of even small amounts of DNA. Absolute quantification enables the determination of the copy number of the given mutation, which is why it is excellently suitable for examining LB samples of melanoma patients. ctDNA analyzed with this method can be used as a predictive biomarker; it enables the measurement of tumor heterogeneity/dynamics and the identification of mutations showing resistance to targeted therapies. It can also be used to investigate the early response to therapy [27,28].

In our study, prospective sampling was carried out to collect LB cfDNA and retrospective sample collection was performed to complement the strength of the statistical analyses. The aims of our study were (i) to determine Breslow depth (BD) and group the samples according to Clark’s classification [29,30,31], (ii) to quantify tumor-derived (td) DNA and cfDNA, (iii) to identify *BRAF* p.V600E somatic mutations not only in the tumor, but also in LB samples, (iv) to compare alterations between histological and LB sample, (v) to determine the diagnostic utility of dPCR technics compared to the *BRAF* 600/601 StripAssay^®^, (vi) to find a correlation between tumor depth, tdDNA, cfDNA, and variant allele frequency (VAF) of *BRAF* p.V600E, and cfVAF, and (vii) to find differences among Clark’s level groups considering the histological and molecular results. For this purpose, histological and IHC analysis, DNA isolation, *BRAF* 600/601 StripAssay^®^, and digital PCR were performed on the pro- and retrospective samples.

## 2. Materials and Methods

### 2.1. Patient Samples and Study Design

For a prospective cohort (*n* = 34), formaldehyde-fixed paraffin-embedded (FFPE) and LB samples were matched, and 68 DNA PCM patient samples were tested. LB was performed before surgical resection. A retrospective cohort including 100 patients with FFPE samples was intended for the digital PCR protocol development and its analytical characterization. A total of 50 FFPE samples were positive for *BRAF* p.V600E, and 50 were negative for this with *BRAF* 600/601 StripAssay^®^.

**Figure 1:**
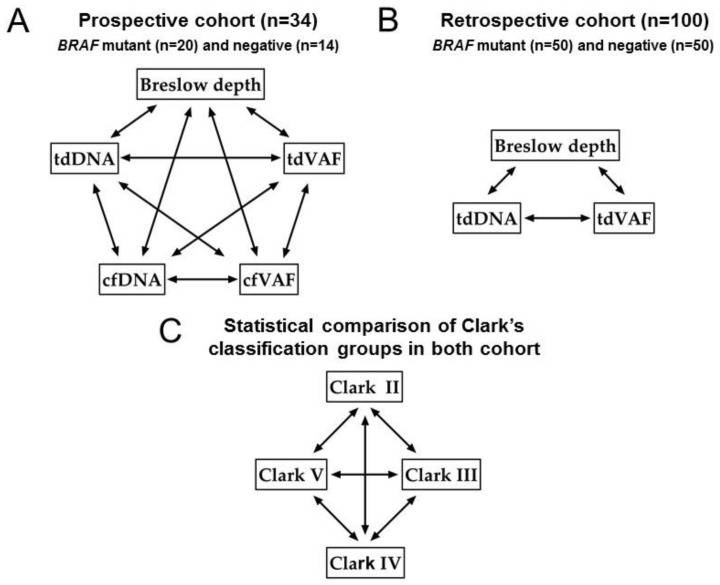
The study design. (**A**): Correlation analyses between tumor Breslow depth (BD), tdDNA, cfDNA, tdVAF of *BRAF* p.V600E, and cfVAF in the prospective cohort. (**B**): Correlation analyses between tumor BD, tdDNA, and tdVAF in the retrospective cohort. (**C**): Kruskal–Wallis statistical comparison of Clark’s level groups BD, tdDNA, cfDNA, tdVAF, and cfVAF in the prospective cohort and BD, tdDNA, and tdVAF in the retrospective cohort, respectively. Peripheral blood was taken before surgery. The correlation analyses and the statistical comparison were performed separately in all cases, in all mutants, and in all negative *BRAF* patients. tdDNA: tumor-derived DNA, cfDNA: cell-free DNA, tdVAF: tumor-derived variant allele frequency of *BRAF* p.V600E mutation, cfVAF: cell-free variant allele frequency of *BRAF* p.V600E mutation.

Breslow’s depth and Clark’s level were used for the classification of the patients. In addition to the histological assignment, we also examined the mutational status (*BRAF* p.V600E mutant or negative) using the dPCR method. Correlation analyses were performed in pro- and retrospective cohorts between tumor BD, tdDNA, cfDNA, tdVAF of *BRAF* p.V600E, and cfVAF. The analyses were applied in all cases, including mutant and negative BRAF patients. Statistical analyses were applied to compare Clark’s levels groups using the above-mentioned parameters. The study design is presented in Figure 1.

All protocols have been approved by the author’s respective Institutional Review Board for human subjects (IRB reference number: IV/8465-3/2021/EKU).

### 2.2. Histopathological and Immunohistochemical Analyses

Expert pathologists examined the slides stained with hematoxylin and eosin (H&E). Samples with more than 20% tumor cells were selected for DNA isolation. The following antibodies were used to differentiate malignant melanoma cases: S100 protein (polyclonal, 1:1000 dilution, Leica Biosystems, Wetzlar, Germany), vimentin (clone V9, 1:200 dilution, Leica Biosystems, Wetzlar, Germany), HMB45 (Human Melanoma Black, clone HMB-45, 1:200 dilution, Dako, Agilent Technologies Company, Santa Clara, CA, USA) and melan-A (clone A103, 1:200 dilution, Dako, Agilent Technologies Company, Santa Clara, CA, USA). In addition, the cell proliferation index was assessed using Ki-67 staining (clone MIB1, 1:200 dilution, Dako, Agilent Technologies Company, Santa Clara, CA, USA).

### 2.3. DNA Isolation

Tumor-derived genomic DNA (tdDNA) was isolated from FFPE tissues using the QIAamp DNA FFPE Tissue Kit (Qiagen, Hilden, Germany) according to the manufacturer’s standard protocol, and genomic DNA (gDNA) was subsequently eluted in 50 µL of elution buffer.

A total of 10 mL peripheral blood samples were collected from each patient in EDTA blood collection tubes. Approximately 5 ± 0.1 mL of plasma was then centrifuged at 16,000× *g* for 10 min to remove cellular residues. Cell-free DNA from peripheral blood (PB) plasma was extracted using the QIAamp Circulating Nucleic Acid Kit (Qiagen, Hilden, Germany) and eluted into 35 µL of elution buffer.

DNA concentrations were quantified using the Qubit dsDNA HS Assay Kit and Qubit 4.0 Fluorometer (Thermo Fisher Scientific, Waltham, MA, USA).

The tdDNA and cfDNA were subsequently purified and concentrated with AMPure XP beads (Beckman Coulter, Brea, CA, USA). The degree of fragmentation before and after purification was analyzed by Agilent Bioanalyzer (Santa Clara, CA, USA).

### 2.4. StripAssay

The analytical sensitivity and specificity of this dPCR protocol was evaluated using a BRAF600/601 StripAssay^®^ from ViennaLab Diagnostics, Vienna, Austria. This assay covers nine clinically relevant mutations in the *BRAF* gene. It is certified for human in vitro diagnostics (IVD). The hybridization strips were aligned according to the standardized layout provided with the reagents to interpret the results, and positive bands were then detected and identified. The limit of detection of the reverse hybridization strip assays is 1% VAF.

### 2.5. Digital PCR Reactions

The mixtures were set up in duplicates with a QIAcuity Probe PCR Kit in Nanoplate 26K 24-well plates (Qiagen, Hilden, Germany). The reactions, containing 10 µL 1× Probe PCR Master Mix, 1.3 µL primer–probe mix, and 20 ng DNA template in 40 µL total volume each, were run in a QIAcuity One instrument (Qiagen, Hilden, Germany) at 95 °C for 2 min, followed by 40 cycles of 95 °C for 15 s (ramp 2 °C/s) and 60 °C for 30 s (ramp 2 °C/s), followed by 98 °C for 10 min. The analysis was carried out using the QIAcuity Software Suite Version 2.1. Fluorescence thresholds for optimal resolution and cloud clustering were selected manually to fit all reactions, constituting 85 and 100 RFU for FAM and HEX, respectively. To enhance the accuracy of concentration measurements, the Volume Precision Factor (VPF), adjusting for tiny variations in nanoplate geometry, was applied as recommended by the manufacturer.

### 2.6. Statistical Analysis

Statistical analyses were performed using GraphPad Prism 9 software. For all pairings, spearman correlation analyses were applied between Breslow depth, tdDNA, cfDNA concentration, tdVAF, and cfVAF. The Mann–Whitney test determined differences between Clark’s level group’s histological and molecular parameters (Figure 1). A value of *p* < 0.05 was considered to be statistically significant.

## 3. Results

### 3.1. Patients Clinicopathological Characteristics

The average patient’s age was 61 in the prospective study (range: 36–100), while 64 was in the retrospective cohort (range: 28–88). The gender distribution was 16/18 and 55/45 male/female in the pro- and retrospective study, respectively. In all cases, the tumor samples were surgically removed before the oncological treatment. In the prospective study, LB was performed before surgical resection.

### 3.2. Histological Features and Molecular Findings

In the prospective patient’s average BD was 4.83 mm (range: 0.09–14.41), tdDNA concentration was 18.75 ng/µL (range: 0.48–59.43), tdVAF of *BRAF* p.V600E was 20.2% (range: 0–88.25), cfDNA concentration was 3.33 ng/µL (range: 0.05–11.43), and cfVAF was 36.55% (range: 0–100). In the retrospective patient’s average BD was 4.27 mm (range: 0–35), tdDNA concentration was 17.39 ng/µL (range: (0.19–53), and tdVAF was 15.33% (range: 0–82.74). Groupings were also made according to mutation status (Table 1).

In both study populations, four Clark classification categories were grouped (Clark II–V, Clark I level was excluded from this study). The above-mentioned average parameters in the different Clark groups are shown in Table 2.

### 3.3. Diagnostical Characterization of the dPCR Analyses

The diagnostical specificity and sensitivity of the developed dPCR-based approach were tested using the tdDNA and cfDNA samples with known *BRAF* p.V600E status based on existing StripAssay^®^ results. The study’s diagnostic sensitivity and specificity were 98.6 and 97%, respectively. In the prospective study, no differences were proven between the two methods (100% specificity and sensitivity). In the retrospective study, the sensitivity was 99%, while the specificity was 98.5%. The diagnostic characterization of the dPCR analyses, including positive and negative predictive values, is presented in Table 3.

The prospective study demonstrated the adequacy of the digital PCR method to determine *BRAF* status, especially for LB. The sensitivity threshold was determined by minimal dilution. The lower limit of detection for the *BRAF* p.V600E variant determined with the use of optimized dPCR protocol was 12 pg of DNA.

### 3.4. Correlation Analyses

In the prospective cohort from all patients, a positive correlation was proved between the BD and tdDNA concentrations (r = 0.54, *p* = 0.001), the BD and cfDNA concentrations (r = 0.86, *p* < 0.0001), tdDNA concentrations and tdVAF of *BRAF* p.V600E (r = 0.76, *p* < 0.0001), tdDNA concentrations and cfVAF (r = 0.59, *p* = 0.0002), and tdVAF and cfVAF (r = 0.65, *p* < 0.0001), while no significant association was found between the BD and tdVAF (r = −0.12, *p* = 0.5), the BD and cfVAF (r = 0.006, *p* = 0.97), tdDNA and cfDNA concentrations (r = 0.07, *p* = 0.7), tdVAF and cfDNA concentration (r = 0.008, *p* = 0.96), and cfDNA concentration and cfVAF (r = 0.13, *p* = 0.47).

If only the *BRAF* p.V600E mutant cases are considered in the prospective study, a positive correlation was proved between the BD and tdDNA concentrations (r = 0.57, *p* = 0.008), the BD and tdVAF (r = 0.47, *p* = 0.03), the BD and cfDNA concentrations (r = 0.76, *p* < 0.0001), the BD and cfVAF (r = 0.47, *p* = 0.03), tdVAF and cfVAF (r = 0.51, *p* = 0.02), and cfDNA concentration and cfVAF (r = 0.73, *p* = 0.0003), while no significant statistical association was found between tdDNA concentrations and tdVAF (r = 0.41, *p* = 0.02), tdDNA and cfDNA concentrations (r = 0.15, *p* = 0.53), tdDNA concentrations and cfVAF (r = 0.07, *p* = 0.77), tdVAF and cfDNA concentration (r = 0.35, *p* = 0.12).

If only the *BRAF* p.V600E negative cases are considered in the prospective study, a positive correlation was proved only between the BD and cfDNA concentrations (r = 0.77, *p* = 0.002). At the same time, no significant association was found between the BD and tdDNA concentrations (r = 0.15, *p* = 0.6), the BD and tdVAF (r = −0.09, *p* = 0.75), the BD and cfVAF (r = 0.01, *p* = 0.97), tdDNA concentrations and tdVAF (r = 0.11, *p* = 0.7), tdDNA and cfDNA concentrations (r = 0.41, *p* = 0.15), tdDNA concentrations and cfVAF (r = −0.27, *p* = 0.35), tdVAF and cfDNA concentration (r = −0.29, *p* = 0.3), tdVAF and cfVAF (r = −0.29, *p* = 0.31), and cfDNA concentration and cfVAF (r = −0.33, *p* = 0.24).

In the retrospective cohort of all 100 patients, a positive correlation was detected between the BD and tdDNA concentrations (r = 0.36, *p* = 0.002), while no significant correlation was proved between the BD and tdVAF (r = −0.04, *p* = 0.7), and tdDNA concentrations and tdVAF (r = −0.06, *p* = 0.52). If only the mutant cases are considered, a positive correlation was proved between the BD and tdDNA concentrations (r = 0.31, *p* = 0.02), the BD and tdVAF (r = 0.37, *p* = 0.009), and tdDNA concentrations and tdVAF (r = 0.33, *p* = 0.018). If only the negative cases are considered, a positive correlation was detected between the BD and tdDNA concentrations (r = 0.33, *p* = 0.02), while no significant association was proved between the BD and tdVAF (r = 0.02, *p* = 0.87), and tdDNA concentrations and tdVAF (r = 0.01, *p* = 0.93).

**Figure 2:**
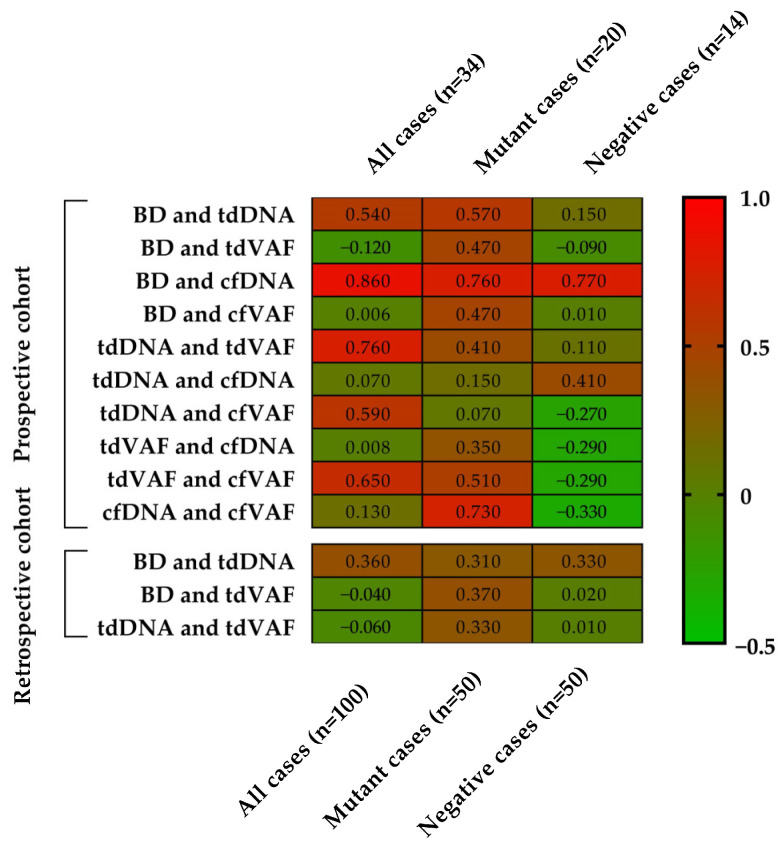
The heatmap results of the Spearman correlation analyses. Spearman correlation coefficients (r) show a color transition from green to red and a corresponding value of −0.5 to 1. BD: Breslow depth, tdDNA: tumor-derived DNA, cfDNA: cell-free DNA, tdVAF: tumor-derived variant allele frequency of *BRAF* p.V600E mutation, cfVAF: cell-free variant allele frequency of *BRAF* p.V600E mutation.

**Figure 3:**
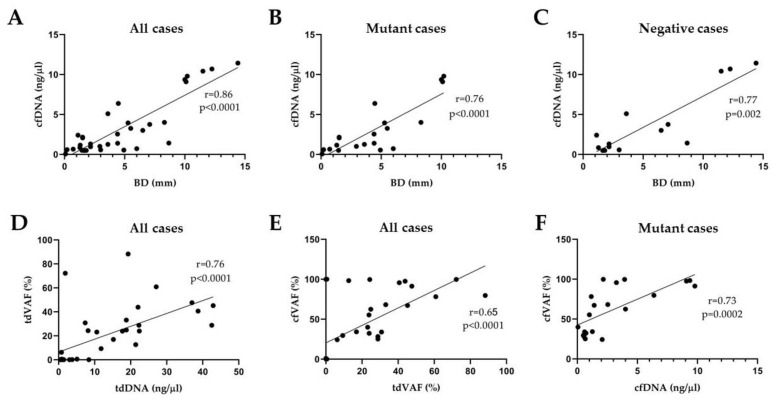
Graphical presentation of the results of correlation analyses in the prospective cohort. A strong positive correlation (r > 0.6) was confirmed between BD and cfDNA concentration in all (**A**), mutant (**B**), and negative (**C**) cases. There is also a strong correlation between the tdDNA concentration and the tdVAF (**D**), between the tdVAF and the cfVAF (**E**) in all cases, and between the cfDNA and cfVAF in mutant cases (**F**). BD: Breslow depth, tdDNA: tumor-derived DNA, cfDNA: cell-free DNA, tdVAF: tumor-derived variant allele frequency of *BRAF* p.V600E mutation, cfVAF: cell-free variant allele frequency of *BRAF* p.V600E mutation.

The correlation analysis results are summarized in Figure 2. An association was detected if the Spearman correlation coefficients were positive with <0.05 *p*-value. The strongest positive correlations (r > 0.6) were confirmed in the prospective cohort. A strong correlation was detected between BD and cfDNA concentration in all mutant and negative cases, between the tdDNA concentration and the tdVAF, between the tdVAF and the cfVAF in all cases, and between the cfDNA and cfVAF in mutant cases (Figure 3).

### 3.5. Statistical Comparison of Clark’s Classification Groups

The results of the Mann–Whitney statistical test between Clark’s level group’s histological and molecular parameters are presented in Table 4. A statistically significant differentiation of BD was found between the Clark II and III (*p* = 0.0131), Clark II and IV groups (*p* = 0.0303), and between the Clark III and V patients (*p* = 0.0063), considering all prospective cases. There is also a significant distinction between the Clark II and V (*p* = 0.0357), Clark III and V (*p* = 0.0048) of tdVAF, and between Clark III and V groups of cfDNA yield (*p* = 0.0170). In the mutant cases, significant differentiation of BD was detected between Clark II and III (*p* = 0.0256). There is also a significant differentiation between the Clark II and III (*p* = 0.0256) and III and IV groups (*p* = 0.0002), considering the cfDNA yield. In the *BRAF*-negative cases, a statistically significant distinction was found between the III and V groups’ BD (*p* = 0.0238).

In the retrospective study, statistically significant differentiation of BD was detected between the Clark II and III groups (*p* = 0.0022), Clark IV and V groups (*p* = 0.0168), and Clark II and IV, II and V, III and IV, and III and V patients (*p* < 0.0001) considering all cases. If only the *BRAF* p.V600E mutant cases are considered, statistically significant differentiation of BD also was detected between II and III (*p* = 0.001), II and V (*p* = 0.0079), IV and V (*p* = 0.0039), II and IV, and III and V groups (*p* < 0.0001), and of tdVAF between the group II and III (*p* = 0.127), II and IV (*p* = 0.0019). If only the negative cases are considered, a significant distinction was proved in BD of groups II and IV (*p* = 0.0069), II and V (*p* = 0.0485), III and IV (*p* < 0.0001), and III and V (*p* = 0.0018), respectively.

## 4. Discussion

Early detection of molecular genetic aberrations in PCM is essential for effective treatment and improved patient outcomes. LB is a non-invasive approach that can be repeated. It offers a potential alternative to conventional surgical sampling for the real-time acquisition of information on the background of molecular aberrations. For early molecular diagnosis in precision medicine, especially in cases such as lung and colorectal adenocarcinoma, the use of PB LB has been introduced [32,33,34,35]. Limited information about the application of LB in melanoma is available in clinical practice because a small amount of cfDNA is derived from it [20,36,37]. Aberrations with extremely low allele frequencies, especially in LB samples, are best identified with dPCR. Apart from being an established method for detecting extremely low allele frequencies, dPCR can quantify target DNA without external standards [27].

The present study investigated correlation and statistical analyses between histopathological staging and molecular aberration of the *BRAF* gene in tdDNA and cfDNA obtained from PCM patients. In the prospective study, a total of 68 plasma and paired tdDNA samples, and in the retrospective cohort, a total of 100 tdDNA samples were analyzed using dPCR and reverse hybridization StripAssay. The Breslow depth and Clark classification were applied to categorize the study population. To the best of our knowledge, the above-mentioned parameters have not yet been studied in such a comparison. The main question of this study was whether there is a correlation between the histological characteristics of melanomas and the quantity (DNA yield) and quality (*BRAF* mutation status) of cfDNA from peripheral blood. In addition, we also investigated the analytical characteristics of a novel dPCR technique for the sensitive detection of *BRAF* p. V600E mutation comparing reverse-hybridization StripAssay. The StripAssay was chosen as the reference method because it has IVD certification. This study provides a reliable tumor tissue-validated home-brew technical basis for the detection of *BRAF* p.V600E somatic mutations using dPCR in PCM patients. The optimized dPCR protocol allows the detection of low copies of the mutant allele with masterly analytical sensitivity. The study’s diagnostic sensitivity and specificity were 98.6 and 97%, respectively. In the prospective study, no differences were proven; in the retrospective study, the sensitivity was 99%, while the specificity was 98.5%. The prospective study demonstrated the adequacy of the digital PCR method to determine *BRAF* status, especially for LB.

The strongest positive correlations were confirmed in the prospective cohort. In many comparisons, we identified a correlation between histological and molecular findings. In the prospective cohort of all patients, a positive correlation was proved between the BD and tdDNA concentrations, the BD and cfDNA concentrations, tdDNA concentrations and tdVAF, tdDNA concentrations and cfVAF, and tdVAF and cfVAF. If only the *BRAF* p.V600E mutant cases are considered in the prospective study, a positive correlation was proved between the BD and tdDNA concentrations, the BD and tdVAF, the BD and cfDNA concentrations, the BD and cfVAF, tdVAF and cfVAF, and cfDNA concentration and cfVAF. If only the *BRAF* p.V600E negative cases are considered in the prospective study, a positive correlation was proved only between the BD and cfDNA concentrations.

The explanation of the strong correlation between BD and cfDNA concentration in all mutant and negative cases is that the deeper the tumor spreads, the more cfDNA from the tumor enters the peripheral circulation due to higher vascular density. If a higher tumor-derived *BRAF* p.V600E VAF is confirmed, it is understandable that cfDNA will also result in a higher VAF. The correlation between DNA concentration and *BRAF* VAF may be the reason that DNA from as many mutant clones as possible was subjected to molecular analysis.

When analyzing the statistical differences between the different Clark level groups’ histological and molecular parameters, a statistically significant differentiation of BD was found between the Clark II and more severe histopathological groups. It is not surprising following the spread of the tumor because the patients with the worst stage were placed in the Clark V category. If only the *BRAF* p.V600E mutant cases are considered, statistically significant differentiation of BD was also detected between Clark’s II and III, II and IV, II and V, III and V, and IV and V. The cfDNA concentration and cfVAF of *BRAF* p.V600E were the highest in the Clark V category. If only the *BRAF* p.V600E mutant cases are considered, there is a significant difference in tdVAF between groups II and III and II and IV. In the mutant cases, significant differentiation was detected between the Clark II, III, III, and IV groups regarding the cfDNA yield, which can also be explained by the fact that cases with a more severe stage classification release more cfDNA into the peripheral circulation.

## 5. Conclusions

Our study provides a reliable tumor tissue-validated technical basis for the detection of *BRAF* p.V600E somatic mutations in PCM patients LB cfDNA. So far, no data have been published for evaluating the association between tumor depth and nucleic acid levels in LB specimens from melanoma. In this study, we also presented a positive correlation between the tumor depth and the yield of cfDNA in PB plasma in all, mutant, and wild-type cases. This observation is also supported by the fact that a statistically significantly higher concentration of cfDNA can be isolated from Clark V category cases compared to the others.

Our results may be a novel approach to the applicability of LB in the early diagnosis of PCM and in monitoring the follow-up of the disease and the response to the therapy. Further clinical studies with higher case numbers are needed to prove the findings described here.

## Figures and Tables

**Table 1:** Average clinicopathological and molecular findings of the study population. Ranges are in parentheses. tdDNA: tumor-derived DNA, cfDNA: cell-free DNA, tdVAF: tumor-derived variant allele frequency of *BRAF* p.V600E mutation, cfVAF: cell-free variant allele frequency of *BRAF* p.V600E mutation.

	Prospective Cohort				Retrospective Cohort			
	Clark II (*n* = 4)	Clark III (*n* = 14)	Clark IV (*n* = 10)	Clark V (*n* = 6)	Clark II (*n* = 9)	Clark III (*n* = 41)	Clark IV (*n* = 37)	Clark V (*n* = 13)
Age (year)	54 (42–66)	59 (42–78)	59 (37–89)	72 (36–100)	62 (35–82)	62 (29–85)	66 (28–83)	66 (50–88)
Breslow depth (mm)	0.2 (0.1–0.2)	3.5 (0.7–10.1)	5.4 (1.5–11.5)	9.1 (2.2–14.4)	1.1 (0.3–2.2)	2.7 (0.1–9.2)	5.1 (1.1–15.3)	9.4 (1.8–35)
tdDNA concentration (ng/µL)	9 (7.4–10.6)	14.4 (0.5–42.9)	15 (0.4–38.7)	6.2 (0.7–21.4)	11.1 (0.4–26.3)	19.5 (1.2–53)	17.7 (1.6–46.8)	19.8 (3.4–49.7)
all tdVAF of *BRAF* p.V600E (%)	26.9 (23.1–30.8)	24.6 (0–72.2)	22.7 (0–88.3)	2.1 (0–12.6)	4.5 (0–14)	18.5 (0–80.5)	15.7 (0–82.7)	12.3 (0–51.6)
mutant tdVAF of *BRAF* p.V600E (%)	26.9 (23.1–30.8)	35.6 (6.2–72.2)	37.8 (9.3–88.3)	12.6	7.2 (0–14)	31.5 (0–80.5)	34.1 (6.2–82.7)	31.8 (0–51.6)
negative tdVAF of *BRAF* p.V600E (%)	–	0.24 (0–0.6)	0.1 (0–0.2)	0 (0–0.1)	0.1 (0–0.2)	0.1 (0–0.6)	1.6 (0–22)	0.2 (0–0.6)
cfDNA concentration (ng/µL)	0.3 (0.1–0.6)	2.3 (0.5–9.1)	3.7 (0.5–10.4)	6.5 (1.4–11.4)	-			
all cfVAF of *BRAF* p.V600E (%)	37 (33.9–39.9)	44.1 (0–99.8)	36.4 (0–95.7)	16.4 (0–98.3)	-			
mutant cfVAF of *BRAF* p.V600E (%)	37 (33.9–39.9)	64.2 (24.3–99.8)	60.4 (29.5–95.7)	98.3	-			
negative cfVAF of *BRAF* p.V600E (%)	–	0 (0–0.1)	0.4 (0–0.9)	0 (0–0.1)	-			

**Table 2:** Average clinicopathological and molecular findings of the study population, including negative wild-type cases using Clark-level categories. The variant allele frequency (VAF) is also broken down according to mutation status. Ranges are in parentheses. tdDNA: tumor-derived DNA, cfDNA: cell-free DNA, tdVAF: tumor-derived variant allele frequency of *BRAF* p.V600E mutation, cfVAF: cell-free variant allele frequency of *BRAF* p.V600E mutation.

	Prospective Cohort			Retrospective Cohort		
	All (*n* = 34)	Mutant Cases (*n* = 20)	Negative Cases (*n* = 14)	All (*n* = 100)	Mutant Cases (*n* = 50)	Negative Cases (*n* = 50)
Age (year)	61 (36–100)	55 (36–78)	71 (45–100)	64 (28–88)	60 (35–82)	67 (28–88)
Breslow depth (mm)	4.83 (0.09–14.41)	4.35 (0.09–10.2)	5.51 (1.12–14.41)	4.27 (0–35)	4.21 (0.1–35)	4.33 (0–15.1)
tdDNA concentration (ng/µL)	18.75 (0.48–59.43)	20.34 (0.8–42.9)	2.06 (0.48–59.43)	17.39 (0.19–53)	14.03 (0.58–45.4)	20.75 (0.19–53)
tdVAF of *BRAF* p.V600E (%)	20.2 (0–88.25)	34.26 (6.23–88.25)	0.11 (0–0.55)	15.33 (0–82.74)	31.16 (0–82.74)	0.74 (0–21.96)
cfDNA concentration (ng/µL)	3.33 (0.05–11.43)	3.02 (0.05–9.79)	3.78 (0.48–11.43)	-		
cfVAF of *BRAF* p.V600E (%)	36.55 (0–99.8)	62.05 (24.27–99.8)	0.12 (0–0.93)	-		

**Table 3:** Diagnostical characterization of the dPCR analyses. The diagnostical utility of the developed dPCR-based approach was tested using the tdDNA and cfDNA samples with known *BRAF* p.V600E status based on existing StripAssay^®^ results.

	All Cases (*n* = 134)	Prospective Cases (*n* = 34)	Retrospective Cases (*n* = 100)
Sensitivity (%)	98.6	100	99
Specificity (%)	97	100	98
Positive predictive value (%)	97.2	100	98
Negative predictive value (%)	98.5	100	99

**Table 4:** Statistical comparison (*p* values) of the different Clark classification categories in both cohorts. Mann–Whitney statistical test showing a significant difference is in bold (*p* < 0.05). BD: Breslow depth, tdDNA: tumor-derived DNA, cfDNA: cell-free DNA, tdVAF: tumor-derived variant allele frequency of *BRAF* p.V600E mutation, cfVAF: cell-free variant allele frequency of *BRAF* p.V600E mutation, n.s.: non-significant.

		Prospective Cohort (*n* = 34)					Retrospective Cohort (*n* = 100)		
		BD	tdDNA	tdVAF	cfDNA	cfVAF	BD	tdDNA	tdVAF
**All cases**	Clark II vs. III	**0.0131**	n.s.	n.s.	n.s.	n.s.	**0.0022**	n.s.	n.s.
	Clark II vs. IV	**0.0303**	n.s.	n.s.	n.s.	n.s.	**<0.0001**	n.s.	n.s.
	Clark II vs. V	n.s.	n.s.	**0.0357**	n.s.	n.s.	**<0.0001**	n.s.	n.s.
	Clark III vs. IV	n.s.	n.s.	n.s.	n.s.	n.s.	**<0.0001**	n.s.	n.s.
	Clark III vs. Clark V	**0.0063**	n.s.	**0.0048**	**0.017**	n.s.	**<0.0001**	n.s.	n.s.
	Clark IV vs. Clark V	n.s.	n.s.	n.s.	n.s.	n.s.	**0.0168**	n.s.	n.s.
**Mutant cases**	Clark II vs. III	**0.0256**	n.s.	n.s.	**0.0256**	n.s.	**0.001**	n.s.	**0.0127**
	Clark II vs. IV	n.s.	n.s.	n.s.	n.s.	n.s.	**<0.0001**	n.s.	**0.0019**
	Clark II vs. V	n.s.	n.s.	n.s.	n.s.	n.s.	**0.0079**	n.s.	n.s.
	Clark III vs. IV	n.s.	n.s.	n.s.	**0.0002**	n.s.	n.s.	n.s.	n.s.
	Clark III vs. Clark V	n.s.	n.s.	n.s.	n.s.	n.s.	**<0.0001**	n.s.	n.s.
	Clark IV vs. Clark V	n.s.	n.s.	n.s.	n.s.	n.s.	**0.0039**	n.s.	n.s.
**Negative cases**	Clark II vs. III	-	-	-	-	-	n.s.	n.s.	n.s.
	Clark II vs. IV	-	-	-	-	-	**0.0069**	n.s.	n.s.
	Clark II vs. V	-	-	-	-	-	**0.0485**	n.s.	n.s.
	Clark III vs. IV	n.s.	n.s.	n.s.	n.s.	n.s.	**<0.0001**	n.s.	n.s.
	Clark III vs. Clark V	**0.0238**	n.s.	n.s.	n.s.	n.s.	**0.0018**	n.s.	n.s.
	Clark IV vs. Clark V	n.s.	n.s.	n.s.	n.s.	n.s.	n.s.	n.s.	n.s.

## Data Availability

The data presented in this study are available on request from the corresponding author. The data are not publicly available to protect the rights of patients.

## References

[1] Abbas O., Miller D.D., Bhawan J. (2014). Cutaneous Malignant Melanoma: Update on Diagnostic and Prognostic Biomarkers. Am. J. Dermatopathol..

[2] Schadendorf D., Fisher D.E., Garbe C., Gershenwald J.E., Grob J.-J., Halpern A., Herlyn M., Marchetti M.A., McArthur G., Ribas A. (2015). Melanoma. Nat. Rev. Dis. Primers.

[3] Thanh D.N.H., Prasath V.B.S., Hieu L.M., Hien N.N. (2020). Melanoma Skin Cancer Detection Method Based on Adaptive Principal Curvature, Colour Normalisation and Feature Extraction with the ABCD Rule. J. Digit. Imaging.

[4] Pavri S.N., Clune J., Ariyan S., Narayan D. (2016). Malignant Melanoma: Beyond the Basics. Plast. Reconstr. Surg..

[5] Takahashi J., Nagasawa S. (2020). Immunostimulatory Effects of Radiotherapy for Local and Systemic Control of Melanoma: A Review. Int. J. Mol. Sci..

[6] Seedor R.S., Orloff M. (2022). Treatment of Metastatic Melanoma in the Elderly. Curr. Oncol. Rep..

[7] Dhomen N., Marais R. (2009). BRAF Signaling and Targeted Therapies in Melanoma. Hematol. Oncol. Clin. N. Am..

[8] Siroy A.E., Boland G.M., Milton D.R., Roszik J., Frankian S., Malke J., Haydu L., Prieto V.G., Tetzlaff M., Ivan D. (2015). Beyond BRAFV600: Clinical Mutation Panel Testing by next-Generation Sequencing in Advanced Melanoma. J. Investig. Dermatol..

[9] Ascierto P.A., Kirkwood J.M., Grob J.-J., Simeone E., Grimaldi A.M., Maio M., Palmieri G., Testori A., Marincola F.M., Mozzillo N. (2012). The Role of BRAF V600 Mutation in Melanoma. J. Transl. Med..

[10] Frisone D., Friedlaender A., Malapelle U., Banna G., Addeo A. (2020). A BRAF New World. Crit. Rev. Oncol. Hematol..

[11] Tímár J., Ladányi A. (2022). Molecular Pathology of Skin Melanoma: Epidemiology, Differential Diagnostics, Prognosis and Therapy Prediction. Int. J. Mol. Sci..

[12] Daum G., Eisenmann-Tappe I., Fries H.-W., Troppmair J., Rapp U.R. (1994). The Ins and Outs of Raf Kinases. Trends Biochem. Sci..

[13] Cutler R.E., Stephens R.M., Saracino M.R., Morrison D.K. (1998). Autoregulation of the Raf-1 Serine/Threonine Kinase. Proc. Natl. Acad. Sci. USA.

[14] Davies H., Bignell G.R., Cox C., Stephens P., Edkins S., Clegg S., Teague J., Woffendin H., Garnett M.J., Bottomley W. (2002). Mutations of the BRAF Gene in Human Cancer. Nature.

[15] Garnett M.J., Marais R. (2004). Guilty as Charged: B-RAF Is a Human Oncogene. Cancer Cell.

[16] Cantwell-Dorris E.R., O’Leary J.J., Sheils O.M. (2011). BRAFV600E: Implications for Carcinogenesis and Molecular Therapy. Mol. Cancer Ther..

[17] Śmiech M., Leszczyński P., Kono H., Wardell C., Taniguchi H. (2020). Emerging BRAF Mutations in Cancer Progression and Their Possible Effects on Transcriptional Networks. Genes.

[18] Wan J.C.M., Massie C., Garcia-Corbacho J., Mouliere F., Brenton J.D., Caldas C., Pacey S., Baird R., Rosenfeld N. (2017). Liquid Biopsies Come of Age: Towards Implementation of Circulating Tumour DNA. Nat. Rev. Cancer.

[19] Siravegna G., Marsoni S., Siena S., Bardelli A. (2017). Integrating Liquid Biopsies into the Management of Cancer. Nat. Rev. Clin. Oncol..

[20] Gaiser M.R., von Bubnoff N., Gebhardt C., Utikal J.S. (2018). Liquid Biopsy to Monitor Melanoma Patients. J. Dtsch. Dermatol. Ges..

[21] Nikanjam M., Kato S., Kurzrock R. (2022). Liquid Biopsy: Current Technology and Clinical Applications. J. Hematol. Oncol..

[22] Kamińska P., Buszka K., Zabel M., Nowicki M., Alix-Panabières C., Budna-Tukan J. (2021). Liquid Biopsy in Melanoma: Significance in Diagnostics, Prediction and Treatment Monitoring. Int. J. Mol. Sci..

[23] Váraljai R., Elouali S., Lueong S.S., Wistuba-Hamprecht K., Seremet T., Siveke J.T., Becker J.C., Sucker A., Paschen A., Horn P.A. (2021). The Predictive and Prognostic Significance of Cell-Free DNA Concentration in Melanoma. J. Eur. Acad. Dermatol. Venereol..

[24] Dube S., Qin J., Ramakrishnan R. (2008). Mathematical Analysis of Copy Number Variation in a DNA Sample Using Digital PCR on a Nanofluidic Device. PLoS ONE.

[25] Quan P.-L., Sauzade M., Brouzes E. (2018). DPCR: A Technology Review. Sensors.

[26] Mao X., Liu C., Tong H., Chen Y., Liu K. (2019). Principles of Digital PCR and Its Applications in Current Obstetrical and Gynecological Diseases. Am. J. Transl. Res..

[27] Whale A.S., Cowen S., Foy C.A., Huggett J.F. (2013). Methods for Applying Accurate Digital PCR Analysis on Low Copy DNA Samples. PLoS ONE.

[28] Thierry A.R., Luthra R., Singh R.R., Patel K.P. (2016). A Targeted Q-PCR-Based Method for Point Mutation Testing by Analyzing Circulating DNA for Cancer Management Care. Clinical Applications of PCR.

[29] Büttner P., Garbe C., Bertz J., Burg G., d’Hoedt B., Drepper H., Guggenmoos-Holzmann I., Lechner W., Lippold A., Orfanos C.E. (1995). Primary Cutaneous Melanoma. Optimized Cutoff Points of Tumor Thickness and Importance of Clark’s Level for Prognostic Classification. Cancer.

[30] Bunnell A.M., Nedrud S.M., Fernandes R.P. (2022). Classification and Staging of Melanoma in the Head and Neck. Oral Maxillofac. Surg. Clin. N. Am..

[31] Puckett Y., Wilson A.M., Farci F., Thevenin C. (2023). Melanoma Pathology. StatPearls.

[32] Sefrioui D., Sarafan-Vasseur N., Beaussire L., Baretti M., Gangloff A., Blanchard F., Clatot F., Sabourin J.-C., Sesboüé R., Frebourg T. (2015). Clinical Value of Chip-Based Digital-PCR Platform for the Detection of Circulating DNA in Metastatic Colorectal Cancer. Dig. Liver Dis..

[33] García-Foncillas J., Tabernero J., Élez E., Aranda E., Benavides M., Camps C., Jantus-Lewintre E., López R., Muinelo-Romay L., Montagut C. (2018). Prospective Multicenter Real-World RAS Mutation Comparison between OncoBEAM-Based Liquid Biopsy and Tissue Analysis in Metastatic Colorectal Cancer. Br. J. Cancer.

[34] Parikh A.R., Leshchiner I., Elagina L., Goyal L., Levovitz C., Siravegna G., Livitz D., Rhrissorrakrai K., Martin E.E., Van Seventer E.E. (2019). Liquid versus Tissue Biopsy for Detecting Acquired Resistance and Tumor Heterogeneity in Gastrointestinal Cancers. Nat. Med..

[35] Esagian S.M., Grigoriadou G.Ι., Nikas I.P., Boikou V., Sadow P.M., Won J.-K., Economopoulos K.P. (2020). Comparison of Liquid-Based to Tissue-Based Biopsy Analysis by Targeted next Generation Sequencing in Advanced Non-Small Cell Lung Cancer: A Comprehensive Systematic Review. J. Cancer Res. Clin. Oncol..

[36] Diefenbach R.J., Lee J.H., Rizos H. (2019). Monitoring Melanoma Using Circulating Free DNA. Am. J. Clin. Dermatol..

[37] Ricciardi E., Giordani E., Ziccheddu G., Falcone I., Giacomini P., Fanciulli M., Russillo M., Cerro M., Ciliberto G., Morrone A. (2023). Metastatic Melanoma: Liquid Biopsy as a New Precision Medicine Approach. Int. J. Mol. Sci..

